# A randomised wait-list controlled clinical trial of the effects of acceptance and commitment therapy in patients with type 1 diabetes: a study protocol

**DOI:** 10.1186/s12912-015-0101-y

**Published:** 2015-11-19

**Authors:** Anna Lindholm-Olinder, Johan Fischier, Jenny Fries, Sven Alfonsson, Veronika Elvingson, Jan W Eriksson, Janeth Leksell

**Affiliations:** Department of Medical Sciences, Clinical Diabetology and Metabolism, Uppsala University, Uppsala, Sweden; Department of Endocrinology, Metabolism and Diabetes, Uppsala University Hospital, Uppsala, Sweden; Department of Public Health and Caring Sciences, Uppsala University, Uppsala, Sweden; Department of Clinical Science and Education, Karolinska Institute, Södersjukhuset, Stockholm, Sweden

**Keywords:** Type 1 diabetes, Acceptance and commitment therapy, Adult

## Abstract

**Background:**

In order to manage the acute and long-term effects of living with a chronic disease such as diabetes, both medical treatment and good psychosocial support are needed. In this study, we wish to examine whether a psychological group intervention targeting people with poorly controlled type 1 diabetes can be helpful in augmenting quality of life while also lowering participants’ HbA1c level. The group intervention will consist of a brief treatment developed from a branch of cognitive behavioural therapy called acceptance and commitment therapy, which is part of the so-called third wave of cognitive behavioural therapy. Common for these third-wave therapies, the focus is less on the content and restructuring of thoughts and more on the function of behaviour. Here, we describe the protocol and plans for study enrolment.

**Methods/Design:**

This on-going study is designed as a randomised wait-list controlled trial. Eighty patients aged 26–55 years and with an HbA1c level >70 mmol/mol at the time of enrolment will be included.

**Discussion:**

In this study, we will assess the effect of starting acceptance and commitment therapy group treatment for patients with type 1 diabetes and its effect on glycaemic control and well-being.

**Trial registration:**

Current controlled trials: ISRCTN17006837, registered 12^th^ January 2015

**Electronic supplementary material:**

The online version of this article (doi:10.1186/s12912-015-0101-y) contains supplementary material, which is available to authorized users.

## Background

The role of nursing, psychology and behaviour medicine in the management of diabetes has gained increasing attention in the past decade, but the field is still young and rather narrow in scope [1–3]. Some of the progress made in diabetes treatment has helped to ease the burden of living with diabetes, while some of the advances have also called for more intensive and demanding self-care [4, 5]. In order to manage both the acute and long-term effects of living with a chronic disease such as diabetes, both medical treatment and good psychosocial support are needed [6].

Among patients with diabetes, psychiatric co-morbidity is associated with poorer glycaemic control [7, 8] and contributes (along with genetic variation and other stress factors) to why so few patients with diabetes reach glycaemic control goals. People with diabetes are two to four times more likely to be diagnosed with depression than those without the disease [4, 9–11]. The repetitive practical and emotional burdens that often accompany the self-management of diabetes play an important role in the increased risk of depression [12].

### Acceptance and commitment therapy

Acceptance and commitment therapy (ACT) is part of the so-called third wave of cognitive behavioural therapy (CBT). Common to these third-wave therapies, focus is less on the content and restructuring of thoughts and more on the function of behaviour. In practise, these therapies focus on teaching patients skills of coping and empowerment, such as strategies of acceptance and mindfulness [13].

The theoretical background of ACT is based on functional contextualistic philosophy of science and modern learning theory, mainly relational frame theory and contextual behavioural science. Both theories strive to understand human behaviour and language from its context and use this knowledge to predict and influence behaviour. ACT can be seen as a unified model of behavioural change, rather than a specific set of methods or techniques [13].

Similar to CBT, ACT acknowledges the relevance of language and cognition in psychopathology and psychotherapy, but ACT does not consider thoughts and beliefs to directly cause other behaviours. Therefore, it is seen as unnecessary to target the content of thoughts in order to promote behavioural change. Instead, thoughts are valued the most for their usefulness in achieving a more valued life [13].

From an ACT perspective, psychological and physical pain is unavoidable and a natural part of the human condition—sickness, death, separation and other large and small disappointments are bound to happen to us if we lead a life that matters to us. While pain might be part of the human condition, suffering does not have to be.

Suffering is considered to be caused by our ability through language to categorise, predict, evaluate, reminisce about the past and worry about things yet to come. Since this stance of accepting pain can be provocative for some patients, it is important in treatment to emphasise that accepting pain is not a passive state but a proactive stance that focuses on behaving in ways that lead to better health and quality of life (QoL).

Accepting the inevitability of pain and struggle, including physical illness and distress, is the first step towards psychological flexibility. The overarching goal in a treatment using ACT is thus to increase patients’ psychological flexibility concerning thoughts, feelings and behaviour. Psychological flexibility is the ability to accept one’s own difficult feelings and thoughts while at the same time striving to live a meaningful life according to one’s personal values [13]. Thus, psychological flexibility involves adjusting behaviour according to the current situation in a helpful manner and not by the ebb and flow of thoughts and emotions. The opposite, psychological inflexibility, can include sticking to a behaviour and mind-set even though it continues to create suffering. Psychological inflexibility is often manifested as emotional avoidance, i.e., the attempt to avoid or ignore unpleasant thoughts, emotions and situations. In ACT, emotional avoidance is seen as the main reason why humans suffer from psychological illness. This emotional avoidance can take many forms, such as avoidance of close relationships, distractions such as excessive game playing, binge eating and the use of drugs or alcohol. These strategies are negatively reinforced as they offer somewhat temporary relief from unpleasant experiences.

Experiential avoidance is not negative per se, but often becomes a problem when it leads to psychological inflexibility and stands in the way of leading a rich and valued life [14].

The ACT model of psychological flexibility is based upon six core processes: acceptance, verbal defusion, mindfulness, self-as-context, values and committed action. In therapy, these processes are targeted through exercises and values clarification. As ACT is a behavioural therapy, the focus lies in experience-based learning, both in-session and as committed actions to try in-between sessions. In summary, experiential avoidance and psychological inflexibility may be important factors in many diabetes patients’ struggle with self-care. Avoidance can make a person ignore recommendations or behave in ways that are not helpful, such as not monitoring blood sugar levels. Inflexibility may manifest as resistance to change and difficulties in adapting to new circumstances. Helping patients to improve in these factors may thus help improve self-care and health.

### Empirical evidence

The current empirical evidence for ACT is rapidly emerging, but is still not as sound as the evidence for CBT. ACT is approved as evidence-based practice for the treatment of depression and chronic pain by the American Psychological Association. Other areas for implementation are also being considered [15] ACT has been tested in numerous behavioural medicine areas such as pain [16], chronic physical diseases [17] and obesity [18, 19], with data showing preliminary good results.

The Swedish institute for assessing health care interventions, SBU, issued a report on diabetes care in 2009 in which they stated that more studies were needed in order to assess the effectiveness of CBT and other interventions for individuals with type 1 diabetes [20]. In a Swedish randomised controlled trial, Amsberg et al. [21] tested the effectiveness of CBT (primarily administered in a group setting) in patients with diabetes. The study specifically aimed at maintaining behaviour change, as previous studies have shown that improvements made directly post-treatment are rarely maintained during follow-up. In the study, the 40 participants in the intervention group significantly improved regarding glycaemic control as measured by HbA1c level, diabetes-related distress, well-being, perceived stress, anxiety and depression, as well as frequency in self-monitoring of blood glucose in comparison to the control group.

To date, there has only been one study testing the effectiveness of ACT in a diabetes population of patients with type 2 diabetes. The study randomly allocated 81 patients to either a control group (in which participants received education about how to manage their diabetes) or to an ACT intervention. Participants in the ACT intervention group received the same education, as well as a 1-day ACT workshop, where the participants were taught how to apply mindfulness and acceptance skills to difficult thoughts and emotions. After 3 months, the participants in the ACT group were more likely to use these coping strategies and reported better diabetes self-care as well as having an HbA1c value in the target range [22].

### Study aim

For this study, we wish to examine whether a psychological group intervention targeting people with poorly controlled type 1 diabetes can be helpful in augmenting QoL while also lowering participants’ HbA1c level. The group intervention will consist of seven sessions of ACT and three follow-up sessions on glycaemic control and well-being.

## Methods/Design

### Study design and setting

The design of this study is a randomised wait-list controlled trial. The study will be conducted at Akademiska sjukhuset, Uppsala University Hospital, Uppsala.

### Participants and recruitment

Eighty patients aged 26–55 years with an HbA1c level >70 mmol/mol at the time of enrolment will be included. Exclusion criteria to the study will be ongoing severe depression, eating disorders or other severe mental illnesses, alcohol or substance abuse, or severe diabetes complications. Oral and written information about the present study will be given to patients who fulfil the inclusion criteria. Written informed consent or assent for participation in the study will be obtained from the participants.

### Randomisation

Participants who fulfil the inclusion criteria and are willing to participate in the study and have completed the pre-test will be randomly allocated to either the intervention or wait-list control group. A flow chart of the study is shown in Additional file 1.

### Intervention

The treatment is scheduled to run over a period of 6 months. Participants in the treatment condition will learn mindfulness and acceptance strategies to cope with diabetes-related distress. The treatment will focus on living according to one’s values and on behavioural activation. As the treatment is targeted towards a population with poorly controlled diabetes, the intervention will also include 1 week of measurements with a hidden continuous glucose monitoring system so that patients can see how their behaviour influences their blood glucose levels. All sessions will be led by a psychologist with specialisation in CBT and training in ACT, and a nurse specialised in diabetes. Each session will have an ACT-specific theme about how to promote psychological flexibility. The topics will include the acceptance of thoughts and feelings, and mindfulness. Exercises in mindfulness will be based in concrete practice, both during the course sessions and as homework. Acceptance will be practiced through experiential exercises, writing exercises and discussion. Each patient will commit to what is important for him or her to focus on in the next follow- up visit. The treatment session protocol is included in Additional file 2.

### Measures and data collection

Before starting ACT we will collect the following data from the participants: HbA_1c_ level, height, weight, insulin requirements, mean frequency of self-monitoring of blood glucose, Hospital Anxiety and Depression scale (HADS), ‘Check your health’ scale, and the Acceptance and Action Questionnaire 2 (AAQ2). These data will also be collected 6 and 12 months after starting ACT.

### HADS

The original HADS, which measures anxiety and depression, was developed by Zigmond and Snaith [23]. The HADS consists of 14 items, of which seven relate to anxiety and seven to depression. Responses are rated on a four-point Likert scale score (0–3), where 0 represents the lowest level of anxiety and depression and 3 the highest level. The Swedish version of HADS has been used in this study, which has been translated by Lisspers et al. [24].

### Check your health

‘Check your health’ measures perceived physical and emotional health, social relationships and general QoL on a vertical thermometer scales, ranging from 0 to 100, with 0 indicating low perceived health and QoL. Using the same scales, a person reports what their perceived physical and emotional health, social relationships and QoL would be if they did not have diabetes. The measured difference between, for example, physical health with and without diabetes is defined as the physical burden of diabetes. When the difference results in a positive value, meaning that, for example, imagined physical health without diabetes is reported to be lower than that with diabetes, the burden is interpreted as zero. In this study, the marginal values for no burden, low burden, high burden or very high burden will be arbitrary. ‘Check your health’ has been tested on adolescents and adults with diabetes and has shown good reliability and validity [25, 26].

### AAQ2

AAQ2 is a self-assessment questionnaire designed to measure psychological acceptance/flexibility and emotional avoidance, and how these two factors affect the individual’s freedom of action. The instrument includes 10 items in the form of statements that the respondent takes a position on in a seven-point Likert scale (1 = never true, 7 = always true), although seven of the 10 items are reversed (2, 3, 4, 5, 7, 8 and 9). Scores are calculated by summing the scores from the 10 items. Higher total values indicate a higher degree of flexibility [27].

### Statistical analysis and power calculation

To detect a difference of 6 mmol/mol in HbA1c (SD ± 11) at least 35 participants are needed in each group (power 80 % and alfa 0.05). In view of the drop-off, 80 patients will be included. The differences between the groups will be analysed with unpaired t-tests with 95 % confidence intervals and *p* < 0.05 considered as significant. Repeated- measures analysis of variance will be used for comparisons in the groups in pre- and post-measuring with the same confidence interval and level of significance. When testing differences between groups regarding AAQ2 and ‘Check your health’, the same statistical analysis will be used.

### Ethical considerations

The ethical review board in Uppsala, Sweden, has approved the study (2014/301).

## Discussion

The aim of this study is to test the effects of an ACT group treatment and its effect on glycaemic control and well-being. ACT is a modern psychological intervention.

Previous studies have reported good preliminary results for ACT in numerous behavioural medicine areas [13, 28–30].

We have chosen to include patients with higher HbA1c values (above 70 mmol/mol) because we want to see if the participants using the tools they receive from ACT treatment can lower their HbA1c level and also experience improved well-being. To detect changes in perceived physical health, general QoL and the burden of diabetes, we will use the HADS, *‘*Check your health’ scale and AAQ2. The intervention will take 7 weeks and perceived QoL will be evaluated at baseline, 6 and 12 months to allow comparisons and to determine any changes.

Sweden has a national register that includes 90 % of all patients. Data from this register show that HbA1c level is increasing every year for patients with type 1 diabetes [31]. This finding highlights the need to take strong measures to help patients achieve optimal glycemic control while maintaining a good QoL, especially as good glycaemic control prevents or postpones complications.

The prevalence of depression is overrepresented in patients with diabetes, which indicates the need for psychological support. Psychiatric co-morbidity is associated with poorer glycaemic control among patients with diabetes [7]. Diabetes is a disease in which self-care is ongoing; therefore, the patient is the most important partner in the team, in collaboration with nurses and clinicians, including a psychologist. A psychologist and diabetes specialist nurse will lead this ACT study.

In a study with similar ambitions to this study, patients with type 2 diabetes had a 1- day training workshop with ACT [22]. After 3 months, these patients were more likely to use these coping strategies, reported better diabetes self-care and had HbA1c values within the target range No studies have been conducted in participants with type 1 diabetes. Thus, there is a lack of evidence in the field, and the intent of this study is to address this gap.

## Additional files

Additional file 1:
**Flow chart of the ACT study.** (DOC 76 kb) Additional file 2:
**Treatment session protocol.** (DOCX 16 kb)
