# Supplementary material for: A randomised wait-list controlled clinical trial of the effects of acceptance and commitment therapy in patients with type 1 diabetes: a study protocol
Source: BMC Nurs. 2015 Nov 19;14:61. doi: 10.1186/s12912-015-0101-y (PMC4653856; doi:10.1186/s12912-015-0101-y)
Supplement: Additional file 1: — Flow chart of the ACT study. (DOC 76 kb) [file 12912_2015_101_MOESM1_ESM.doc]

Analyses: unpaired t-test, ANOVA

Analyses: Unpaired t-test, ANOVA

Control group

n= 40

Intervention group

n=40

Randomization

Usual care

Intervention

ACT – start education

**At start:**

HbA1c, height, weight, insulin requirement, mean frequency SMBG Check, your health, Swe-DES 23 and Diabetes treatment satisfaction

**At 6 months:**

HbA1c, height, weight, insulin requirement, mean frequency of SMBG, Check, your health, Swe-DES 23 and Diabetes treatment satisfaction

**At 12 months:**

HbA1c, height, weight, insulin requirement, mean frequency of SMBG, Check, your health, Swe-DES 23 and Diabetes treatment satisfaction counting

**Inclusion criteria**: Age between 26 to 55 years old and have an HbA1c level **>70 mmol/l** at the time of inclusion.

**Exclusions criteria**: Ongoing severe depression, eating disorder or other severe mental illness, alcohol or substance abuse or severe diabetes complication.

The population consists of 383 (out of 1295) who fulfilled the inclusions criteria.

Follow-up session

ACT – start

Follow-up session

Follow-up session

**Additional file 1: Flow chart of the ACT study**
