# Supplementary material for: A randomised wait-list controlled clinical trial of the effects of acceptance and commitment therapy in patients with type 1 diabetes: a study protocol
Source: BMC Nurs. 2015 Nov 19;14:61. doi: 10.1186/s12912-015-0101-y (PMC4653856; doi:10.1186/s12912-015-0101-y)
Supplement: Additional file 2: — Treatment session protocol. (DOCX 16 kb) [file 12912_2015_101_MOESM2_ESM.docx]

Additional file 2

Treatment session protocol

Session 1

Theme: Introduction to the treatment and the burden of diabetes

The introduction to the treatment and to acceptance and commitment therapy (ACT) will include: 1) practical issues regarding the treatment and its structure; 2) a thorough introduction to the behavioural model and the theories and principles behind ACT; 3) a review of how diabetes may affect thoughts and feelings and the potential failure of a control agenda; and 4) an introduction of mindfulness as a strategy to handle thoughts and feelings with a short exercise as a home assignment.

Sessions 2–3

Theme: Acceptance and values

These sessions will include a continued discussion of control strategies and an introduction of acceptance as an alternative. Topics will include an introduction to values as a tool to guide behaviour, discussion and exercises regarding values and everyday behaviour, and continued exercises about mindfulness and values work as home assignments.

Sessions 4–5

Theme: To live according to values

These sessions will include continued discussion of values and everyday behaviour. Topics will include an introduction to verbal defusion and avoidance patterns, and discussion, exercises and assignments regarding mindfulness and valued living.

Session 6

Theme: Defusion and compassion

This session will include continued discussion of verbal defusion. The session will include the following: 1) an introduction to self-observation and exercises in verbal defusion; 2) an introduction about self-compassion and how to behave according to values regarding self and others; 3) discussion about difficult life situations and decisions; and 4) assignments with exercises on mindfulness and self-compassion.

Session 7

Theme: Summing up previous topics

In this session, all the previous themes and discussions will be summarised and previous exercises will be repeated. The session will include a discussion of how to use new skills in everyday life, and evaluation and feedback from participants.
